# Supplementary material for: Are carbon emissions trading and green financial instruments synergistic? -Comprehensive quantitative research based on content analysis
Source: PLoS One. 2024 Mar 7;19(3):e0298601. doi: 10.1371/journal.pone.0298601 (PMC10919601; doi:10.1371/journal.pone.0298601)
Supplement: S1 Data — (ZIP) [file pone.0298601.s001.zip › Data/地级市Z_F工作报告.zip!/╔╜╢1⁄2/║╩╘≤╩╨/└┤╫╘╣1⁄2╓┌║┼í╛┬φ┐╦╩2╛▌═°í┐_2004.docx]

各位代表:

现在,我代表市人民政府向大会作政府工作报告,请予审议,并请市政协委员和其他列席人员提出意见。

一、2003年工作回顾

过去的一年是我市历史上极不平凡的一年。上半年抗击非典,下半年抗洪救灾。面对突如其来的严重自然灾害,面对异常艰巨的发展任务,全市人民在市委的正确领导下,高举邓小平理论伟大旗帜,躬身实践“三个代表”重要思想,认真贯彻落实党的十六大、十六届三中全会和省市委工作会议精神,解放思想,与时俱进,开拓创新,扎实苦干,全力推进改革开放和现代化建设,国民经济和各项社会事业都有了新的发展,较好地完成了市十六届人大一次会议确定的各项目标任务。抗击非典和抗洪救灾取得重大胜利。在抗击非典这场没有硝烟的战争中,针对我市地处四省交界,外出务工人员多,防范难度大的严峻形势,全市上下众志成城,群防群控,实现了来菏人员不漏查、堵住源头不输入的目标。在抗洪救灾工作中,市委领导靠前指挥,各级政府精心组织,广大军民协同作战,社会各界积极救助,确保了受灾群众的生命安全,确保了黄河大堤的安全,灾区群众生活得到了妥善安置。通过积极的生产自救,灾害损失降到了最低程度。在严重自然灾害面前,全市人民表现出来的巨大凝聚力、坚强战斗力和无私奉献精神,是我们战胜各种困难、争取更大胜利的宝贵精神财富。国民经济持续健康发展。在抗击非典和抗洪救灾的同时,牢牢抓住经济发展不放松,全市经济增长速度不断加快,经济运行质量和效益明显提高。全市完成国内生产总值290亿元,比上年可比增长12.3%;地方财政收入15.96亿元,增长19.8%;城镇居民人均可支配收入5891元,增长5.7%;年末金融机构各项存、贷款余额326.7亿元和292.6亿元,分别比年初增加54亿元和51.4亿元。经济结构调整步伐加快。三次产业比由上年的44∶31∶25调整到40∶36∶24,二三产业比重提高了4个百分点。农业结构进一步优化,粮经作物比由上年的49.5∶50.5调整到44.3∶55.7;林业生产取得历史性突破,新增造林面积200万亩;畜牧业总产值51亿元,增长17.2%。工业经济实现了速度效益同步增长,全市规模以上工业企业完成增加值51.3亿元,增长34.9%;实现利税12.3亿元,增长21.7%;工业经济效益综合指数比上年提高8.92个百分点;高新技术产品销售收入占比提高1.23个百分点。第三产业发展活力不断增强,全社会消费品零售总额141.7亿元,增长14.7%。对外开放水平不断提高,完成进出口总额3.1亿美元,增长85.2%,其中出口2.9亿美元,增长87.5%;合同利用外资2.2亿美元,吸收外商直接投资1.3亿美元,均增长1.8倍。民营经济快速发展,全市注册个体私营企业11.8万户,增长30.6%;从业人员37.1万人,增长51.4%;上缴税金5.3亿元,增长40.3%。固定资产投资实现重大突破。全社会固定资产投资完成162亿元,比上年增长83%;投资过千万元的项目489个,比上年增加269个,其中过亿元的38个,增加15个。鲁花10万吨花生油、百隆4万锭精梳、青啤5万吨扩建、五得利面粉、都庆多功能肽、步长制药、睿鹰化工等一批骨干项目相继竣工投产;东明石化自备电厂、健民制药、呈祥电器、圣达纺织、印龙纺织、长江纺织等一批技改项目即将投产;龙固矿井、达驰智能变压器、黄海粮油、大亚高密度板、广源铜带、东明石化催裂化、天宇泡沫镍、晨鸣20万方中密度纤维板、巨鑫源芦笋综合利用等一批投资过亿元的在建工业项目进展顺利。园区基础设施投入完成12.8亿元,进区项目370个,其中过亿元的项目15个,完成投资57亿元。城市和基础设施建设成效明显。高起点进行第三轮城市总体规划,初步方案已通过专家预审;认真整顿土地市场秩序,城市土地经营实现新突破,完成政府土地收益6.46亿元,其中市区3.5亿元;加大城市开发建设力度,完成投资12亿元,在加快供排水体系和治污工程等基础设施建设的同时,开工建设了国际会展中心、东西城区路网、环城公园等一批重点项目,在建城市住宅小区24个,竣工面积36万平方米;切实加强城市管理,城市面貌明显改观。同时,大力推进基础设施建设,济菏高速公路前期工作全面完成,菏关高速公路顺利开工,国省道和农村道路改造、东鱼河南北支和鄄郓河等骨干河道治理、通信设施建设、电网建设和配电网改造等方面都取得了较好成绩。各项社会事业全面发展。就业和社会保障取得重大突破,市内二、三产业新增就业32万人,市外劳务输出75万人,为13万名下岗职工推荐了一次再就业岗位,5万多名下岗职工实现了再就业;全市企业养老保险费征缴率达到95%,比上年增收5962万元;离退休人员养老金全部实现了社会化按时足额发放,91397名城镇困难群众享受到最低生活保障,初步实现了就业和社会保障的良性循环。取得市级以上重大科技成果91项,科技对经济发展的贡献率不断提高。中小学危房改造力度进一步加大,“普九”水平有所提高,中高等教育资源的整合步伐加快,菏泽学院建设进展顺利。疾病预防控制的长效机制初步建立,公共卫生体系建设得到加强,公立医院产权制度改革取得突破。计划生育管理体制改革初见成效。国土资源管理和环境保护工作明显加强。精神文明创建活动广泛开展。文化大市建设取得新的进展。兵役及双拥工作取得新的成绩。普法教育扎实推进,社会治安综合治理不断深入。体育、广播电视、新闻出版、人防、气象、地震、旅游、档案、史志、民族、宗教、外事、侨务、老龄、妇女儿童和残疾人等项事业都有新的发展。政府自身建设进一步加强。深入学习“三个代表”重要思想,广泛开展新一轮解放思想大讨论,形成了团结一致干事业、齐心协力促发展的生动局面。认真践行“约法三章”,坚持务实勤政,不做表面文章;依法行政,不失职渎职;廉洁从政,不以权谋私。深化行政审批制度改革,审批项目由原来的1287项减为401项,精减了68.8%;实行政务公开,先后设立了市长公开电话、政策法规咨询中心和政府网站,增强了政府工作的透明度;推进财政综合改革,加强行政效能监察和审计监督,加大“三乱”治理力度,全市经济发展环境逐渐好转。各位代表!过去的一年我们遇到的困难比预料的大,取得的成绩比预料的好,成绩确实来之不易。这是市委正确决策、坚强领导的结果,是人大、政协监督支持的结果,是全市人民团结拼搏的结果。实践使我们更加深刻地体会到,只要我们全心全意地贯彻执政为民的根本宗旨,真心实意为广大人民群众办实事、办好事,在人民群众生命财产安全受到威胁、切身利益受到侵害时,各级干部能够挺身而出,就能赢得广大人民群众的拥护和支持,就没有克服不了的困难;只要我们坚持不懈地把发展作为第一要务,切实转变作风,真抓实干,不论在任何情况下都能牢牢抓住发展不放松,就能推动全市经济和社会事业更快更好地发展;只要我们始终如一地自觉维护市委的领导权威,自觉接受人大、政协和社会各界的监督,自觉密切与工、青、妇等群团组织和各民主党派的联系,就能在全市上下形成团结合作干事业、一心一意谋发展的强大合力;只要我们坚定不移地从菏泽实际出发,创造性贯彻执行党的路线方针政策,紧紧围绕建设“一个中心、两大基地”,认真做好调整结构、扩大投资、优化环境三篇文章,就能瞄准经济工作的主攻方向,实现菏泽发展的新跨越,不断把菏泽的现代化事业推向新的胜利。为此,我代表市人民政府,真诚地向各民主党派、人民团体、无党派爱国人士,向驻菏部队和武警官兵,向人大代表、政协委员,向全市各族人民群众,向所有关心支持菏泽发展的国内外朋友表示衷心的感谢!尽管2003年全市经济和社会发展取得了很大成绩,但是与全省的差距仍在继续拉大,在我们前进的道路上仍面临着比全省更加突出的困难、矛盾和问题。不但经济总量小、结构不合理的矛盾仍然非常突出,机制性、体制性障碍远没有从根本上消除,而且基础设施的瓶颈制约依然相当严重。不但财源基础薄弱,财政收支矛盾还十分尖锐,而且就业压力大,社会保障能力差,弱势群体和部分受灾群众的生产生活还有很多困难。尤其是农业产业化水平低,抗御自然灾害的能力差,去年的洪涝灾害造成全市农业直接经济损失高达50亿元,尽管我们采取了一系列促进农民增收的措施,全市农民人均纯收入却比上年减少86元,仅为2212元,进一步拉大了与全省的差距。从政府工作的角度看,治理经济环境的成效还不够显著,全市的投资发展环境还不够宽松,特别是各级政府的服务效率和驾驭市场经济的能力还需要进一步提高。对这些矛盾和问题,我们一定高度重视,采取有力措施,认真加以解决。

二、2004年国民经济和社会发展的目标任务和工作安排

2004年是省委、省政府实施“突破菏泽”战略的第一年,也是我市抢抓机遇,乘势而上,跨越发展的关键一年。做好今年的工作,意义特别重大。根据省委、省政府的部署和市委的安排,今年全市经济和社会发展的总体要求是:以邓小平理论和“三个代表”重要思想为指导,全面贯彻落实党的十六大和十六届三中全会精神,按照省委、省政府提出的“三三一一”目标要求,牢固树立全面、协调、可持续的发展观,以加快发展为主题,以结构调整为主线,以扩大投资为总抓手,以优化环境为关键措施,高度重视解决“三农”问题,大力推进工业化、城市化进程,加快服务业发展,促进国民经济持续快速协调健康发展和社会全面进步。主要预期目标是:国内生产总值增长14%,二三产业比重提高3个百分点;地方财政收入增长13%;全社会固定资产投资增长50%;社会消费品零售额增长15%;进出口增长33%,实际利用外资增长37%;农民人均纯收入和城镇居民人均可支配收入分别增长8%和6%;新增城镇就业6万人以上,城镇登记失业率控制在3.8%以内;人口自然增长率控制在7‰以内。实际工作中要加倍努力,力求发展得更快更好。为此,市政府拟着重抓好以下十项工作:

(一)以招商引资为重点,千方百计扩大投资。扩大投资是实现菏泽经济超常规、高速度、跨越式发展的关键之举。必须继续把扩大投资作为全市经济工作的总抓手,以招商引资为重点,确保固定资产投资突破240亿元。拓宽投资来源渠道。从菏泽经济欠发达、资本积累少的实际出发,必须把招商引资作为扩大投资的主渠道,最大限度地吸引国内外企业到我市直接投资。同时,进一步优化银企关系,加强银企合作,争取国有商业银行的大力支持。充分利用国家继续实施积极财政政策和省扶持欠发达县的政策,千方百计争取国家和省政策性投资。降低门槛,放宽政策,搞好服务,激活民间资本,扩大民间投资。通过畅通各种融资渠道,促进投资的大突破,经济的大发展。加大项目策划力度。重点围绕“一个中心、两大基地”建设项目策划和产业策划,增强招商引资项目的产业关联度。着眼于培植主导产业,努力提高项目策划质量,加大项目推介力度,增强投资吸引力,形成产业招商新优势。改进招商引资方式。进一步建立和完善招商引资的激励机制,在充分调动全社会招商引资积极性的基础上,面向市内外、国内外聘请专门人才,造就一支高水平的专业招商队伍,构筑招商引资网络。继续抓好以商招商、登门招商、网上招商和中介招商,强化招商引资效果。重点面向日韩、港台及长江三角洲、珠江三角洲等国家和地区大规模地组织开展招商活动。同时,采取切实措施办好国际牡丹花会和林产品交易会,提高招商引资水平。强化投资载体建设。严格执行耕地保护政 策,引导工业项目集中布局,坚定不移地走工业园区化的路子,举全市之力加快园区经济发展。以市开发区为重点,营造服务效率最高、投资成本最低、管理运作最规范的新型机制。坚持“政府办区、企业办园”路子,鼓励支持有条件的企业和单位进区办园。加强园区基础设施建设,提高产业配套和服务配套水平,形成产业集中、特色鲜明的园区发展新格局。改善投资发展环境。在加快菏关高速公路建设,争取济菏高速公路尽快开工,做好德商、菏徐等高速公路前期准备工作,努力改善基础设施条件的同时,进一步增强全社会的投资服务意识,下大气力营造良好的投资服务环境。进一步完善行政审批服务中心运行机制,全面实行“一个窗口对外”、“一站式服务”。进一步完善服务承诺制,建立健全对政府部门服务经济发展绩效的考评机制,增强各级各部门为投资服务的自觉性。继续重拳出击纠风治乱,对顶风而上、执法犯法的害群之马严惩不贷,斩断向投资者吃拿卡要的“黑手”。坚持除恶务尽,硬起手腕,深挖严打,从重从快处理破坏投资环境的不法分子。全面推行部门预算,继续深化财政改革,铲除“三乱”滋生土壤,在全社会形成亲商、安商、富商、护商的良好氛围。

(二)坚持城乡统筹,加大“三农”工作力度。认真落实胡锦涛总书记来我市视察时的讲话精神,把解决“三农”问题作为政府全部工作的重中之重。按照统筹城乡经济社会发展的要求,坚持“多予、少取、放活”的方针,促进农民增收、农业增效、农村经济社会全面发展。进一步调整农业结构。加大农业科技推广力度,提高粮食综合生产能力,确保粮食单产提高、总产稳定。大力发展瓜菜、林果、花卉等优质、高产、高效农业,促进特色农产品向优势产区集中。充分利用黄河滩区、故道区、沙化区及各类非耕地植树造林,高标准建设国家批复立项的200万亩速生丰产林工程,努力把菏泽建设成为全国平原速生丰产林基地。注重发挥“一牛两羊”的品牌优势,积极发展畜牧养殖小区,推行标准化生产,加快建设与国际接轨的畜牧养殖基地。大力发展农业产业化经营。重点围绕畜牧、林业、果蔬花卉三大产业,完善加工体系,拉长产业链条。加大对龙头企业的扶持力度,着力培育一批辐射作用大、带动能力强的龙头加工企业,力争年内农产品加工龙头企业发展到50家,其中省级龙头企业10家、国家级2家。建立多样化的利益连接机制,完善“公司+基地+农户”模式,把分散的农户与市场连接起来,切实保护好农民利益。积极推进农村城镇化。坚持城镇化与工业化相统一,搞好城镇规划,把县城及中心镇建设与工业小区建设结合起来,强化生产要素的集聚功能,尽快形成各具特色的城镇发展格局。运用市场手段,鼓励引导社会资本参与城镇基础设施和公益事业建设,探索建立多元化的城镇投融资机制。改革户籍管理制度,打破城乡壁垒,以宽松的政策吸纳人口、吸引资金、聚集产业,实现以城带乡,城乡互动,共同发展。加大农村基本建设投入。加快推进村村通柏油路工程,尽快实现村村通客车。加快农村饮水解困工程建设,加大水利设施配套及水毁工程修复力度,集中搞好黄河标准化堤防和滩区安全建设。把农村中小学校舍的维护、改造和建设纳入社会发展和基础设施建设规划,列入政府预算,加快危房改造步伐。扩大农村劳动力转移。牢固树立“只有减少农民,才能富裕农民”的思想,把劳务经济作为一大产业来抓。在积极调整农村经济结构,加快发展农村二三产业,更多吸纳农村劳动力就业的同时,下大力搞好农村劳动力输出服务网络建设,强化就业信息、技术培训、咨询推介等相关服务,促进农村劳动力向城市转移、向市外转移。尤其是要充分发挥各类大中专院校的作用,重点打造我市保安、家政、园艺三大劳务品牌。加大对损害农民工权益案件的查处力度,依法帮助农民工解决工资拖欠问题。继续深化农村改革。认真落实农村税费改革政策,在取消农业特产税的基础上,按规定取消公益金,降低农业税率。健全完善农民负担监督管理机制,加强农村财务管理,加大涉农案件查处和责任追究力度,逐步减轻农民税费负担。在稳定家庭联产承包责任制的基础上,按照“依法、自愿、有偿”的原则,完善农村土地承包政策,创新土地流转机制,促进农村土地有序流转,适度集中。深化粮食流通体制改革,在放开粮食市场的基础上,建立农产品市场流通体系,把对流通环节的间接补贴改为对农民的直接补贴。积极稳妥地推进农村信用社改革,加大金融支农工作力度。建立健全农村社会化服务体系。在进一步完善农业科技推广体制的同时,依托和整合现有供销、农机、农技、灌溉等服务组织,积极发展农产品、农村消费品连锁经营,为农民提供各种产前、产中、产后信息和技术等相关服务。支持农民发展多种形式的农村专业合作组织,努力提高农民的自我服务能力和参与市场竞争的组织化程度。

(三)大力培植骨干企业和支柱产业,积极推进工业化进程。牢牢坚持“全党抓经济,重点抓工业”的方针,大力实施“工业兴市”战略,努力提高工业在国民经济中的比重,促进全市经济结构的战略性调整。以国有资本退出为重点,进一步深化企业改革特别是市属企业改革。坚持优质资产通过嫁接、改造等形式率先退出,劣质资产通过破产、出售等形式强行退出的原则,对国有及国有控股、参股企业进行摸底排查,因企制宜,逐个制定落实国有资本退出措施。依照《公司法》建立起规范的企业法人治理结构,促进企业管理体制和经营机制转变,从根本上启动企业内部活力。实施重点企业带动战略。筛选30家市场前景好、发展潜力大、辐射带动能力强的骨干企业,实行重点扶持,实现企业规模的快速膨胀。力争年内利税过千万元的企业达到40家,其中过5000万元的5家,过亿元的3家。大力发展能源化工产业。重点围绕能源化工基地建设,积极推进煤电化一体化开发进程。加快龙固矿井建设进度,做好彭庄、郭屯、赵楼、郓城矿井及坑口电厂的前期准备工作,搞好菏泽电厂三期工程建设。加快东明石化集团的技术改造和结构调整,支持圣奥、睿源、单县有机化工等化工企业和睿鹰制药、鲁抗药业、步长制药等医药企业的发展,促其迅速膨胀成为支撑能源化工基地的新亮点。提升改造传统优势产业。加大纺织服装、食品加工、木材加工等传统产业技改力度,提高产业技术水平和综合效益。以郓城棉纺群体、天香毛纺、曹县百隆、银河纺织、成武银翔等企业为龙头,加快纺织业产业升级步伐;以菏泽黄海粮油、裕鲁、华瑞、盈享,曹县银香伟业、巨鑫源,东明五得利,定陶九发、鲁花,巨野亚龙、麟州,郓城富士达,单县百寿坊等企业为龙头,加快优质绿色食品产业发展;以定陶林盾,成武森利,曹县曹普、庄寨光明家俱,菏泽大亚、锦江等企业为龙头,形成集高中密度板、桐杨木拼板、条木工艺品及高档家俱为一体的林产品加工产业群。加快发展区域特色经济。按照特色产业、中小企业、专业市场三位一体、互为依托、配套推进的发展思路,立足实际,突出特色,大力发展县域特色经济。对形成一定规模且发展前景广阔的特色经济区域,在水、电、路等基础设施建设等方面给予重点支持,并帮助指导搞好产业配套,使其尽快形成小商品、大市场,小企业、大群体,小资本、大聚集的区域经济发展新格局。

(四)以商贸流通为重点,加快服务业发展步伐。紧紧围绕建设商贸流通中心的战略目标,依托我市区位交通优势,大力实施“商贸兴城”战略,推进服务业快速发展。加快专业批发市场建设。结合新一轮城市总体规划,搞好专业批发市场规划布局。加快施乐购物中心、康庄乐园大市场、三信山海物流中心、恒盛物流中心、牡丹商业广场等大型商业设施建设步伐,增强花都商埠、康庄服装市场和曹县桐木、鄄城中药材、成武大蒜等专业市场的辐射带动能力。大力发展现代物流业。鼓励支持大中型工业企业剥离购销、运储等物流业务,引入市场竞争机制,实行社会化服务。重点发展第三方物流,加快现有仓储、运输、货代、联运企业的改组改造,促其向现代物流企业转型。充分发挥我市区位优势和口岸、铁路、公路等交通运输设施日趋完备的有利条件,积极引进国内外著名物流企业。抓好以菏泽城区为中心的连锁经营,推动其向宽领域、大范围、多行业延伸发展。加快华瑞和德农农资等物流中心建设,力争年内完成县级网络建设,逐步覆盖全市城乡并向周边地区延伸。全面推进旅游、社区服务、房地产开发和金融保险等新型服务业发展。在加快曹州牡丹园改造和郓城宋江武校特色旅游项目建设的同时,着手策划巨野金山旅游区、定陶仿山旅游区和鄄城孙膑故里旅游区等一批旅游项目,运用市场手段,实行招商开发。借助周边旅游资源,加大我市旅游产品推销力度,科学编制旅游线路,力争把牡丹花会纳入全省、全国旅游销售网络,促进全市旅游业的快速发展。加快城镇社区服务体系建设,拓宽服务领域,尤其要大力发展晚间经济,拓宽购物、餐饮、文化、娱乐、健身、休闲等各类服务业的发展空间。进一步简化房产交易手续,激活住房二级市场。推进金融保险业发展,建立健全信用担保体系,引导商业银行扩大授权授信,加大对地方经济发展的资金支持。注重发展信息、信托、证券、会展、拍卖等现代服务业,规范发展会计、审计、律师、公证、监理、资产评估等中介组织。更新思想观念,实行政策扶持,为服务业发展创造一个良好的社会环境。

(五)扩大出口规模,增强参与国际竞争的能力。抓住国家出口退税政策调整的机遇,优化出口产品结构,提高出口产品质量档次,加快实现由初加工产品向精深加工产品转变。坚持扬长避短、发挥优势的原则,重点搞好木制品、纺织服装、果蔬、裘皮、医药化工和工艺人发等六大出口产业的培植。对出口规模较大的企业,在贴息贷款、出口退税等方面,加大政策扶持力度,优先支持出口退税抵押贷款。力争年内有20家企业出口超过500万美元,其中6家企业超过1000万美元。加大国际市场开拓力度。充分利用省委、省政府出台的支持经济欠发达县加快发展的相关优惠政策,积极组织企业有针对性地到国内外参加会展活动,巩固扩大传统市场,大力开拓欧美、非洲和俄罗斯市场,争取出口的国家和地区超过100个。坚持“两头在外”,大力发展加工贸易,扩大原油、大豆、木材、棉花等原料进口和加工产品出口,进一步提高利用国外资源和市场的能力。同时,积极实施“走出去”战略,鼓励支持有条件的企业到国外兴办企业、承揽工程,开展经济技术合作。加快菏泽口岸迁址改造建设进度,争取尽快投入运营,努力拓展周边地区货源空间,使之真正成为区域性的集装箱运输中心。

(六)努力激活民间资本,推动民营经济快速发展。民营经济是推动全市经济快速发展的活力之源,希望所在。必须进一步解放思想,加大措施,全力推动民营经济更快更好地发展。牢固确立民营经济的市场主体地位。放宽准入领域,确保民营经济在投融资、税收、土地、对外贸易等方面与公有制经济享受同等待遇。鼓励支持民营企业特别是市外优势民营企业,以买断、控股、参股、合作、联营等方式参与基础设施和公益事业的投资和经营,参与国有、集体企业的改制、改组和改造,以充分发挥其在繁荣经济、活跃市场、扩大就业、增加税收、参与国企改革等方面的重要作用。切实加大对民营经济的支持力度。积极提供法律、政策、信息、技术、管理等方面的咨询和服务,及时帮助解决资金、场地、水电、交通等方面的困难和问题。尽快建立民营企业信用担保体系,加大金融机构的信贷支持。鼓励引导民营企业搞好体制创新、管理创新、技术创新,建立现代企业制度,提高经营管理水平。支持引导民营企业通过重组联合和技术改造,膨胀规模,提升档次,真正成为推动全市经济发展的主导力量。继续鼓励机关事业单位人员离岗创业,支持全社会所有具有投资经营能力的人更加大胆放手地发展民营经济,真正使那些为发展民营经济做出突出贡献的有识之士在政治上有荣誉、社会上有地位、经济上得实惠。

(七)坚持“四高”方针,加快城市化进程。区域经济发展需要中心城市的带动,实施“突破菏泽”战略有赖于中心城市率先突破。从全市经济社会持续快速协调健康发展的大局出发,必须继续坚持高起点规划、高标准建设、高水平管理、高效益经营的“四高”方针,推进菏泽区域中心城市的率先发展。加快城市规划编制进度。抓紧完善报批第三轮城市总体规划,深化分区详细规划和近期建设规划,加快编制路网、水系、绿地、管网等专业规划,提高控制性详细规划覆盖率。坚持“阳光规划”,完善专家和公众参与机制,提高规划决策的科学化、民主化水平。切实维护规划的统一性和严肃性。加大城市基础设施建设力度。进一步完善城区路网,搞好路政设施配套,全面实行城市区化交通。加快引黄供水、污水处理、牡丹园改造、环城公园等项目建设,开工建设火车站广场、城东南水区、赵王河公园、垃圾处理场等新项目。加大旧城开发和城中村改造力度,新建一批设计水平高、规模大、功能全的居民小区,改善居住环境。全面实施“绿、亮、清”工程,大规模实现城区绿化,高水平实现城市亮化,高质量实现水体变清。切实加强城市管理。坚持建管结合,创新城市管理机制。继续深化环卫体制改革,把城区垃圾收集、清运和处理全部纳入市场化经营轨道。实行城市综合执法,切实解决城市管理中重复执法、多头执法问题。搞好市容综合整治,对窗口部位、中心地带和居民小区进行集中治理,对占道经营、乱倒垃圾、乱摆摊点、乱停车辆、乱贴乱挂等进行专项治理,全面提升城市管理档次。进一步搞好城市经营。以土地经营为重点,走“以城建城、以城养城、以城兴城”的路子,完善城市经营机制,提高城市经营水平。坚持政府高度垄断城市土地一级市场,适度从紧供应土地,形成竞争用地的市场环境,实现土地价值和政府收益最大化。搞好城市节点开发,带动城市土地升值,促进新一轮城市开发建设。进一步整顿土地市场秩序,严格规范经营性土地招标、拍卖、挂牌出让,加大土地出让金和地租征收力度,防止土地收益流失。全市土地经营收益力争达到6亿元。大力推进城市基础设施、公用事业和无形资产经营,拓宽城市建设投融资渠道,形成城市资本“投入、增值,再投入、再增值”的自发展机制。

(八)深化财政改革,提高财税管理水平。按照“量入为出、收支平衡”的原则,推行部门预算,确保所有政府性资金和收支项目全部纳入财政统一管理;建立健全国有资产管理、监督和运营机制,使所有国有资产全部纳入市场化经营轨道,所有财政性收益全部上交财政;推行政府支出管理体制改革,将政府购买性支出全部纳入政府采购,实行“阳光作业”;加快推行国库集中支付制度,统一集中支付所有财政性支出;统筹乡镇预算内外收入管理,按照部门综合预算的办法管住管好乡镇财政。发挥审计职能,强化审计监督,使全市财政管理纳入制度化、规范化运行轨道。强化税收征管。按照“依法征税,应收尽收,坚决不收过头税,坚决制止和防止越权减免税”的要求,强化税源监控,实现税收征管的日常化、规范化。建立健全综合治税体系和工作机制,落实地方税收综合治税考核奖励办法,实现税源信息资源共享,提高税收征管效率。严格支出管理。坚持勤俭节约、依法理财。改进财政支出分配管理方式,规范财政支出范围,优化财政支出方向。从严控制“人、车、会、话”等一般性支出,清理压缩不合理支出,集中资金确保市、县、乡三级工资及时足额发放,确保社会保障支出和农业、科技、教育、公共卫生等重点支出需要。用好财政杠杆。更好地发挥财税政策导向作用和国有资本的吸附引导作用,利用国家财政政策,拓宽财源建设渠道,着力培植优势产品、支柱企业,带动财源结构调整,促进财政收入不断增长,形成财政与经济的良性循环发展格局。

(九)统筹兼顾,全面发展各项社会事业。认真贯彻公民道德建设实施纲要,加强公民道德教育,提高全民整体道德水平。广泛开展多种形式的精神文明创建活动,提高城乡文明程度,争创省级文明城市。实施“科教兴菏”和“人才强市”战略。加快科技成果的引进和利用,鼓励发明和创新,扩大企业自主知识产权拥有量,提高科技对经济发展的贡献率。认真落实农村基础教育“以县为主”的管理体制,继续巩固“普九”成果;推动各类职业教育资源整合,鼓励社会力量办学,促进民办教育快速发展;加快发展高等教育,搞好菏泽学院配套建设。牢固树立人才资源是第一资源的观念,多层次、多渠道、大规模开展人才培训,培养造就适应现代化建设需要的各类人才。创新工作机制,完善激励政策,努力营造有利于各类人才干事创业的社会环境。推进生态市建设。坚持人与自然和谐发展,切实转变生产发展方式、经济增长方式和社会消费方式,努力把菏泽建设成为经济繁荣、环境优美、社会文明的生态城市。加强生态建设和保护,合理开发水、土地、矿产、林木等资源,提高资源利用效益。严格执行建设项目环境影响评价和“三同时”制度,严禁新上污染项目。加强重点污染源治理,广泛推行清洁生产。搞好城市环境综合整治。加快各县城污水处理厂建设。重视农业和农村污染防治,提高人居环境质量。加快计划生育、卫生、文化、体育等各项社会事业发展。全面落实计划生育基本国策,积极推进计划生育综合改革和优质服务,加强流动人口管理,稳定低生育水平,优化出生人口性别结构,不断提高人口素质。加强公共卫生体系建设,健全疾病预防控制体系、突发公共卫生事件医疗救治体系、卫生监督体系和爱国卫生体系;加快市县两级疾病预防控制中心和传染病医院建设;积极推进公立医院产权制度改革,支持民营医疗机构发展;改善乡村医疗卫生条件。围绕建设文化大市,深化文化体制改革,大力发展文化产业,鼓励精品创作,繁荣文化市场,丰富广大群众精神生活。加大体育设施投入,广泛开展全民健身活动,提高全民健康水平和竞技体育水平。积极做好军转干部、退役士兵安置和双拥工作,增进军政军民团结。重视统计工作,确保统计数字的真实性和准确性。贯彻民族宗教政策,促进民族团结。关心支持妇女、儿童、残疾人事业。继续做好广播电视、档案、史志、外事、侨务、老龄、人防、气象、防震减灾、无线电管理等工作,促进社会事业全面发展。

(十)坚持把群众利益放在首位,切实关心群众生产生活。抓住人民群众最现实、最关心、最直接的困难和问题,为群众诚心诚意办实事,尽心竭力解难事,坚持不懈做好事,切实维护好群众利益。千方百计扩大就业再就业。坚持劳动者自主择业、市场调节就业和政府促进就业的方针,积极开发就业岗位。支持下岗职工自主创业、自谋职业、灵活就业。立足发展经济扩大就业,加快劳动密集型产业、民营经济、中小企业和服务业发展,拓宽就业空间。努力改善就业环境,全面落实就业再就业政策,强化社区就业管理和服务职能,确保在48小时内为所有求职者推荐一次就业岗位。完善社会保障体系。搞好“两个确保”和“三条保障线”的衔接,健全养老、医疗、失业、工伤、生育保险和城市居民最低生活保障制度。强化企业养老保险扩面征缴工作,加大欠费清收力度,努力实现企业养老保险全覆盖和企业离退休人员管理服务社会化。进一步做好城市低保工作,实现动态管理下的应保尽保和城市最低生活保障金社会化按月发放。积极推进农村养老保险和“失地农民”养老保险工作。推行以大病统筹为主的新型农村合作医疗制度,扩大医疗保险覆盖面,健全医疗保障体系。实施“扶贫济困”工程。强化政府救灾救济职能,增加对预防保健、基本医疗、义务教育和社会弱势群体救助等方面的支出,建立完善对低收入者和困难家庭的社会救灾、救济、互助、福利体系。进一步搞好灾区重建和经济适用住房、廉租住房、周转住房建设,切实解决城乡困难群体的住房问题。加快建立完善新型农村救济制度,对农村特困户、五保户等救济对象发放“两证一卡”,实行定期定量救济,使他们能够得到最基本的生活保障。健全完善法律援助体系,依法保障弱势群体的合法权益。继续整顿规范市场经济秩序。重点抓好直接关系人民群众生命健康安全的食品、药品市场秩序的专项整治。依法严厉打击制售假冒伪劣商品等违法犯罪行为,规范市场监管,维护经营者和消费者合法权益。加强社会信用体系建设,营造市场公平竞争、各界守法经营、居民放心消费、客商乐于投资的社会环境,塑造诚信菏泽的良好形象。切实维护社会稳定。深入开展“平安菏泽”创建活动,强化治安防控体系建设,推进社会治安综合治理。做好矛盾纠纷排查调处工作,预防群体性事件发生。坚持“重心下移”,切实抓好信访工作。深入开展严打整治斗争,严厉打击刑事犯罪和社会黑恶势力,依法惩治邪教组织。全面贯彻“安全第一、预防为主”的方针,突出抓好易燃易爆、交通、消防和危险化学品、食品卫生管理。认真落实“一岗双责”等安全生产责任制,大力开展专项整治活动,杜绝各类重大恶性事故的发生。各位代表!政府是依法而设、因公而设、为民而设,执政为民是政府的根本宗旨。目前由于我市经济条件所限,部分群众的生产生活困难还难以在短期内全部解决,但是只要我们满怀对人民群众的深厚感情,旗帜鲜明地依法保护人民群众的合法权益不受侵犯,真心诚意地实行政务公开、促进社会公正,坚持不懈地为人民群众办好事、办实事、解难事,尽全力保一方平安、富一方百姓、主一方公道、建一方文明,就一定能够得到广大人民群众的理解和支持!

三、加强政府自身建设的主要措施

面对全市经济社会发展的艰巨任务,各级政府必须按照为民、务实、清廉的要求,进一步加强政府自身建设,不断提高政府工作水平。完善决策机制,增强执政能力。建立健全政府系统科学民主决策制度,突出抓好重大事项集体决策、专家咨询、社会公示和社会听证制度的完善,凡是与群众利益密切相关的决策事项,最大限度地提高透明度和公众参与度,努力提高科学决策的能力。把改革的力度、发展的速度和社会可承受的程度统一起来,统筹兼顾,协调好各方面的关系,努力提高总揽全局的能力。防止主观主义和片面性,对前进中出现的新情况、新问题,坚持用发展的办法加以解决,对苗头性和局部性的问题,善于见微知著,举一反三,及时采取果断措施,防止演变为全局性问题,努力提高科学判断形势的能力。坚持以创新观念为先导,创新思路、创新体制、创新机制,时刻保持清醒头脑,把加快发展的良好愿望与科学求实的态度结合起来,把长远规划与分步实施结合起来,把大胆创新与遵纪守法结合起来,努力提高驾驭市场经济的能力。分析解决矛盾和问题,充分考虑前因后果、相关因素,既对上级负责也对下级负责,既对现实负责也对长远负责,既对局部负责也对全局负责,防止处置不当,引发群体性事件和公共突发事件,努力提高应对复杂局面的能力。转变政府职能,提高行政水平。坚持实行政企、政事、政社分开,继续改进经济调节和市场监管方式,更加重视履行社会管理和公共服务职能,切实把政府的经济管理职能转到主要为市场主体服务和创造良好发展环境上来。按照精简、统一、效能的原则,深化行政体制改革,降低行政成本,提高服务效率,建立政府提速长效机制。实行“阳光政务”,坚持政务公开,健全完善市长公开电话、政府网站、政策法规咨询中心和行政效能投诉中心的运行机制,积极构筑便民利民服务渠道,自觉接受群众监督。坚持依法行政,建设法治政府。以贯彻执行《行政许可法》为契机,强化各级政府及其工作人员在法律规定范围内活动的自觉性,确保行政行为主体合法、程序规范、适用得当、体现公平。认真清理现行有关行政许可的规范性文件,凡是与《行政许可法》不一致的自7月1日起一律停止执行。理顺行政执法体制,规范完善城市相对集中行政处罚权工作。强化行政执法监督,切实做到有权必有责、用权受监督、侵权要赔偿。严格行政执法责任制和行风评议考核制度,完善行政复议制度,规范行政执法行为,严肃查处政令不畅、效率低下和自由裁量失当等问题。加强作风建设,推进反腐倡廉。大力弘扬求真务实精神,大兴求真务实之风,力戒官僚主义、形式主义和光说空话、不干实事的恶劣作风,禁止搞任何形式的“政绩工程”、“形象工程”,坚决反对欺上瞒下、弄虚作假、虚报浮夸行为。坚持政策与实际的统一,从政策调整中抢抓机遇,在贯彻落实上搞好“结合”,把着力点放在抓调研、抓机遇、抓落实上,放在鼓实劲、干实事、求实效上。特别是把政策措施落实作为转变作风的关键环节,建立完善决策目标、执行责任、考核监督“三个体系”,实行严格的目标责任管理,把全年经济社会发展目标及相关政策措施细化分解落实到各级各部门,一级抓一级,层层抓落实,切实把各项工作落到实处,抓出实效,干出水平。同时制定更加科学合理的考核办法,严格兑现奖惩,真正建立起奖勤罚懒、奖优罚劣、论功行赏、赏罚分明的激励约束机制。严格落实廉政建设责任制,完善监督、约束、防范机制,深入开展廉政建设和反腐败斗争。严肃查办以权谋私、侵害群众利益的案件,加大对各类违法违纪行为特别是大案要案的查处力度,坚决惩治清除腐败分子,对侵犯公民权利、侵害群众利益的人和事,一经发现,严惩不贷;对执法犯法、徇私枉法、欺压百姓的害群之马,坚决绳之以法,绝不姑息!

各位代表,目前我市正处在加快发展的关键时期,机遇千载难逢,发展时不我待。让我们紧密团结在以胡锦涛为总书记的党中央周围,在省委、省政府和市委的坚强领导下,认真实践“三个代表”重要思想,紧紧围绕全面建设小康社会的宏伟目标,团结依靠全市人民,解放思想、抢抓机遇、凝聚力量、干事创业,为实现菏泽的超常规、高速度、跨越式发展而努力奋斗!
